# Recent Advances in Anticancer Activity of Novel Plant Extracts and Compounds from *Curcuma longa* in Hepatocellular Carcinoma

**DOI:** 10.1007/s12029-022-00809-z

**Published:** 2022-03-14

**Authors:** Nighat Gull, Fareeha Arshad, Gowhar A. Naikoo, Israr Ul Hassan, Mona Zamani Pedram, Arif Ahmad, Alaa A. A. Aljabali, Vijay Mishra, Saurabh Satija, Nitin Charbe, Poonam Negi, Rohit Goyal, Ángel Serrano-Aroca, Mazhar S. Al Zoubi, Mohamed El-Tanani, Murtaza M. Tambuwala

**Affiliations:** 1 School of Sciences, Maulana Azad National Urdu University, 32, Hyderabad, TS India; 2 Department of Biochemistry, Aligarh Muslim University, U.P., India; 3 Department of Mathematics and Sciences, College of Arts and Applied Sciences, Dhofar University, Salalah, Sultanate of Oman; 4 College of Engineering, Dhofar University, Salalah, Sultanate of Oman; 5 Faculty of Mechanical Engineering-Energy Division, K. N. Toosi University of Technology, P.O. Box: 19395-1999, No. 15-19, Pardis St., Mollasadra Ave., Vanak Sq., Tehran, 1999 143344 Iran; 6 Department of Pharmaceutics & Pharmaceutical Technology, Yarmouk University, Irbid, 21163 Jordan; 7 School of Pharmaceutical Sciences, Lovely Professional University, Phagwara, Punjab India; 8 Department of Pharmaceutical Sciences, Rangel College of Pharmacy, Texas A&M University, Kingsville, TX 78363 USA; 9 School of Pharmaceutical Sciences, Shoolini University of Biotechnology & Management Sciences, Solan, 173229 India; 10 Biomaterials & Bioengineering Lab, Centro de Investigación Traslacional San Alberto Magno, Universidad Católica de Valencia, San Vicente Mártir, 46001 Valencia, Spain; 11 Department of Basic Medical Sciences, Faculty of Medicine, Yarmouk University, Irbid, Jordan; 12 Pharmacological and Diagnostic Research Centre, Faculty of Pharmacy, Al-Ahliyya Amman University, Amman, Jordan; 13 School of Pharmacy & Pharmaceutical Sciences, Ulster University, Northern Ireland, Coleraine, BT52 1SA County Londonderry UK

**Keywords:** Cancer, Curcumin, Hepatocellular Carcinoma, Anticancer

## Abstract

**Purpose:**

Among all forms of cancers, hepatocellular carcinoma (HCC) is the fifth most common cancer worldwide. There are several treatment options for HCC ranging from loco-regional therapy to surgical treatment. Yet, there is high morbidity and mortality. Recent research focus has shifted towards more effective and less toxic cancer treatment options. Curcumin, the active ingredient in the Curcuma longa plant, has gained widespread attention in recent years because of its multifunctional properties as an antioxidant, anti-inflammatory, antimicrobial, and anticancer agent.

**Methods:**

A systematic search of PubMed, Embase and Google Scholar was performed for studies reporting incidence of HCC, risk factors associated with cirrhosis and experimental use of curcumin as an anti-cancer agent.

**Results:**

This review exclusively encompasses the anti-cancer properties of curcumin in HCC globally and it’s postulated molecular targets of curcumin when used against liver cancers.

**Conclusions:**

This review is concluded by presenting the current challenges and future perspectives of novel plant extracts derived from C. longa and the treatment options against cancers.

## Introduction

**Fig. 1:**
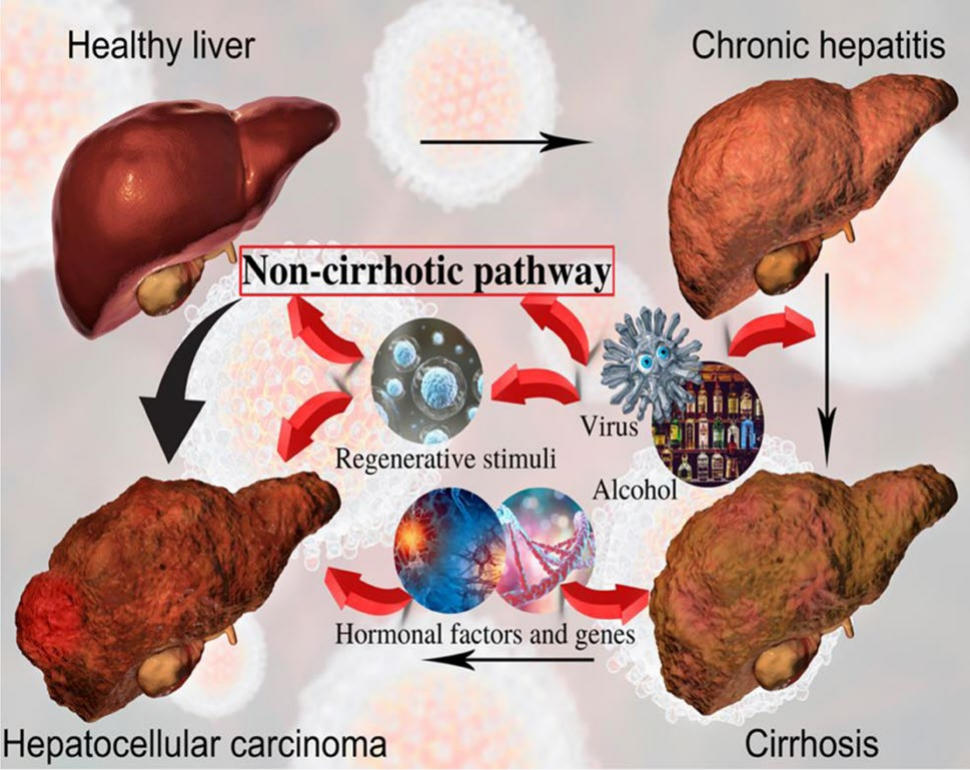
Pathobiology of hepatocellular carcinoma

Hepatocellular carcinoma (HCC) is a primary tumor of the liver and ranks among the fifth most common cancers globally. It is the third leading cause of cancer-related death after lung, colorectal, and stomach cancers [1]. The HCC includes 75–85% of all primary liver cancers and is a significant concern worldwide as it kills half a million people annually around the world [2–4]. In 2021, 42,230 HCC-related cases were recorded worldwide that showed 29,890 in men and 12,340 in women [5]. Research has shown that sub-Saharan Africa and Eastern Asia with Magnolia demonstrate the highest age-standardized incidence rates (ASIR) for HCC (more than 20 per 100,000 individuals). In contrast, South Central Asia, followed by Central and Eastern Europe and Western Asia (less than 5 per 100,000 people) [6–8]. The HCC incidence has increased from 3.1 to 5.1 per 100,000 people in the United States (US) between 1996 and 2006, while liver cancer mortality increased from 3.3 to 4.0 per 100,000 people [9]. The HCC rate has escalated annually by 2–3%, primarily due to factors including cirrhosis, hepatitis C virus (HCV), and non-alcoholic steatohepatitis (NASH) [10, 11]. The developmental pathway of HCC is described in Fig. 1.

Among the many factors, chronic hepatitis virus (HBV) is responsible for over 50% of all global cases [12]. Apart from conventional chemotherapy, several curative protocols such as liver transplantation and surgical resection are used to treat HCC [13]. However, owing to late diagnosis and poor response rates, these surgical and systemic treatments are often unsuccessful in providing survival benefits and improving mortality rates [14]. Up to 80% of patients experiencing HCC recurrence were observed within 5 years [15]. As a result, developing successful and safe therapeutics to increase the effectiveness of HCC therapy is critical. Unfortunately, current therapies have not been proven to be devoid of side effects during prolonged treatments [16]. Side effects, including but not limited to gastrointestinal toxicity, hepatotoxicity, cardiotoxicity, and neurotoxicity, are widespread among patients undergoing chemotherapies and radiotherapies against cancers, including HCC [17]. Therefore, the ongoing quest for anticancer agents derived from plants has played a crucial role in determining how to make chemotherapy safer and reduce its repercussions.

Traditional medicine has contributed many novel therapeutic compounds for preventive and curative treatment compared to other drug discovery sources. Over several decades, scientists analyzed nearly 200 combinations of different anticancer drugs. Half of these drugs come from natural products and their derivatives, which are safer and biologically advantageous [18, 19]. In addition, due to their structural diversification, secondary metabolites like flavonoids, terpenes, vitamins, oils, among others, have been proven to possess antimutagenic and anticancer properties [20, 21]. Among these, the natural phenolic compound has curcumin has anti-inflammatory properties that make it an ideal candidate for treating such chronic diseases [22]. Multiple tumor studies have displayed chemopreventive evidence of this potential therapeutic agent on HCC in human hepatoma cells. Different pathways have been studied that show the mechanism of action of curcumin, which includes prevention of tumor division, metastasis, and triggering apoptosis [23–25]. However, its low bioavailability and short lifetime in the human body is a significant concern. Studies have been done to functionalize curcumin with nanomaterials and other formulations like micelles, microspheres, emulsions, liposomes, and nanogels. In a recent study by Matias and colleagues, it was observed that phytosomes have a considerable potential to be used along with natural drugs, like curcumin, to improve their biocompatibility in the body and therefore effectively aid in the treatment of many inflammatory disease conditions, including cancers [26, 27].

## Statistical Aspects of Hepatocellular Carcinoma Across the World in Particular India, USA, and GCC

Hepatocellular carcinoma has an annual fatality ratio (mortality-to-incidence ratio) of 0.92, the highest reported for a human cancer worldwide [1, 28]. In most countries, men have 2 to 3 times more HCC incidence and mortality than women. The most considerable incidence rates are found in underdeveloped nations [29]. However, this type of cancer is rampant in Eastern Asia, South-East Asia, Northern Africa, and Southern Africa [30]. Liver cancer is among the main reasons for death in Mongolia, Thailand, Cambodia, Egypt, and Guatemala. HCC is caused by chronic infection with the hepatitis B virus (HBV) or the hepatitis C virus (HCV), aflatoxin-polluted foods, excessive alcohol consumption, smoking, type 2 diabetes, and obesity [31]. Geographically, the critical risk factors differ significantly. Fatal HBV infection, aflatoxin exposure, or both are the critical determinants in high-risk HCC infection areas include China, the Republic of Korea, and sub-Saharan Africa [32]. Table 1 shows the number of new liver cancer cases and the estimated deaths in different global regions.

**Table 1:** Trends in the number of new liver cancer cases and estimated deaths due to liver cancer in 2020. (Globocan 2020 from World Health Organization, accessed on 16 July 2021)

World region	Estimated new cases of liver cancer (2020)			Estimated Deaths due to liver cancer (2020)		
	Both sexes	Males	Females	Both sexes	Males	Females
Eastern Africa	12,326	7225	5101	11,542	6718	4824
Middle Africa	6072	4204	1868	5716	3956	1760
Northern Africa	31,913	20,468	11,445	30,352	19,408	10,944
Southern Africa	2601	1626	975	2447	1574	873
Western Africa	17,630	11,619	6011	16,887	11,084	5803
Caribbean	3383	1928	1455	3182	1808	374
Central America	11,819	5895	5924	11,231	5584	5647
South America	24,293	13,662	10,631	23,153	12,963	10,190
Northern America	46,599	32,505	14,094	34,818	23,021	11,797
Eastern Asia	491,687	357,292	134,395	449,534	326,903	112,631
South-Eastern Asia	99,265	71,496	27,769	95,668	69,084	26,584
South-Central Asia	54,698	36,213	18,485	52,769	34,973	17,796
Western Asia	11,342	6998	4344	10,927	6737	4190
Central and Eastern Europe	24,782	15,184	9598	23,002	13,985	9017
Western Europe	26,128	18,506	7622	23,657	16,420	7237
Southern Europe	24,796	16,938	7858	21,243	14,446	6797
Northern Europe	11,924	7451	4473	10,513	6493	4020
Australia and New Zealand	3344	2429	915	2503	1713	790
Melanesia	933	572	361	911	560	351
Polynesia	58	41	17	55	38	17
Micronesia	84	68	16	70	54	16
Low human development index	33,097	20,742	12,355	31,602	19,756	11,846
Medium human development index	99,994	69,180	30,814	95,859	66,358	29,501
High human development index	548,935	391,686	157,249	524,307	373,738	150,569
Very high human development index	223,321	150,489	72,832	178,107	117,470	60,637
World	905,677	632,320	273,357	830,180	577,522	252,658

**Fig. 2:**
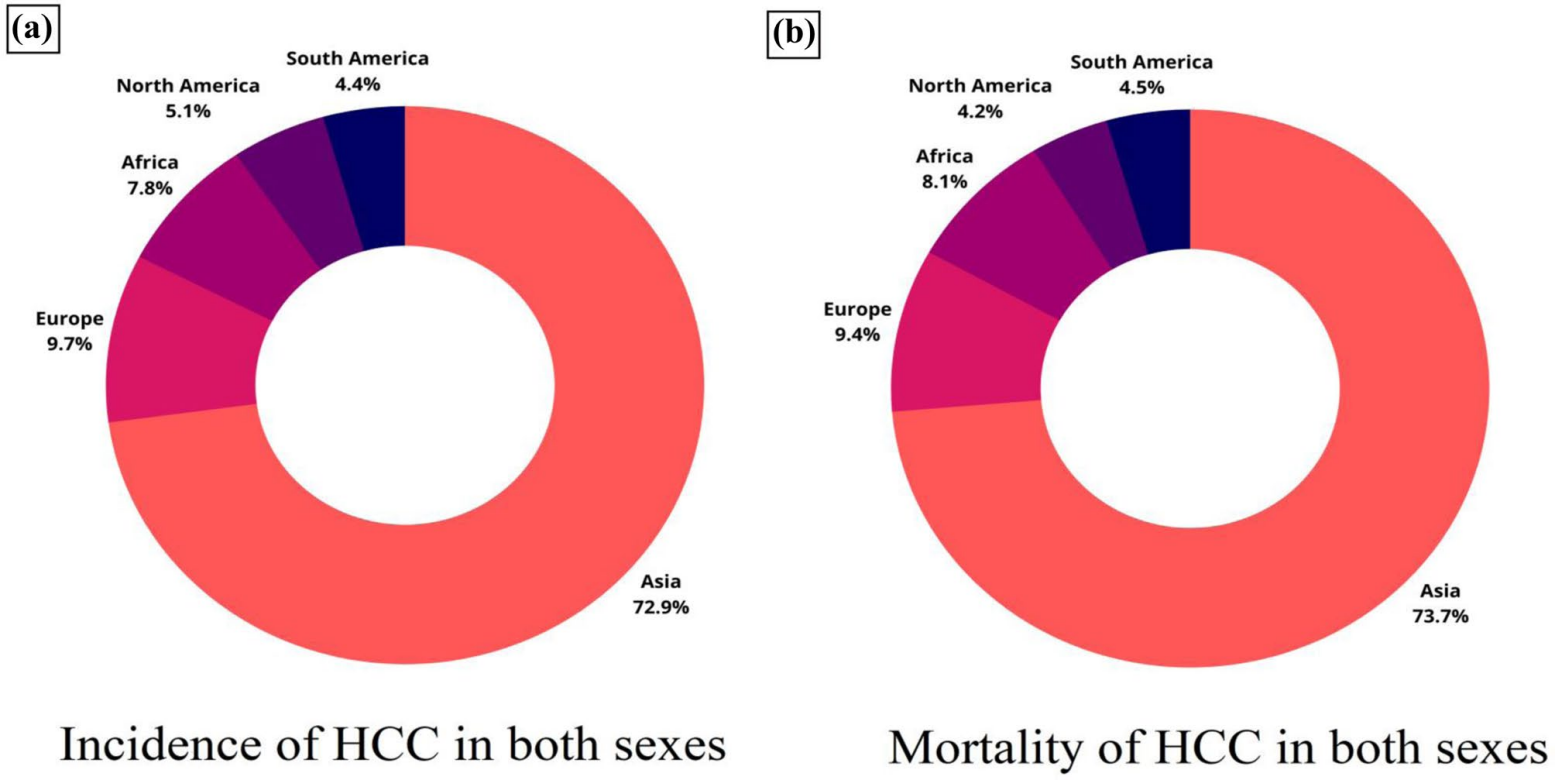
Trends in HCC cases across different global regions in 2018. **a** The pie chart shows the distribution of HCC for both sexes. **b** The pie chart shows the mortality cases primarily due to HCC. Data were taken with permission from [36], copyright@2019 (WCRJ)

However, HCV infection may be the primary reason in other nations like Japan, Italy, and Egypt. Since the late 1970s, rates and mortality of HCC have decreased in many high-risk countries in Eastern and South-Eastern Asia and Japan. Since 1995, Italy’s rates have also been reduced [6, 33]. These patterns are most likely due to lower HBV and HCV seroprevalence in the population and lower aflatoxin exposure [34]. In 2015, it was reported that 290 million people worldwide had chronic infections that were usually asymptomatic and that many infected people went undiagnosed [35]. In high-risk countries like East Asia, the possible effect of HBV vaccination has been a significant public health achievement and thus has successfully helped reduce the number of HBV infection cases and HCC cases [36]. Even though non-viral risk factors also contribute to liver cancers, eliminating viral hepatitis remains the most crucial strategy for preventing HCC cases worldwide. HBV infection accounts for 56% and HCV infection for 20% of liver cancer deaths, respectively [30]. This can be understood from Fig. 2, which reveals the global trend in the number of HCC cases in 2018.

By the end of 2019, compulsory HBV vaccination was launched for babies among 189 member states, with about 85% of infants receiving three vaccine doses worldwide [37]. However, the countries with the maximum rates of HCV infection are primarily low and middle-income countries, where most instances of diseases occur because of unhygienic injections and other inappropriate methods [38]. Therefore, a key element of HCV management is increasing safety initiatives to control infection, such as transfusion screening, precautions in transmittance through intimate relations like a mother to child, the availability of sterile needles, and control of infections in health care utilities [38].

### India

There is a lack of nationally representative data, so we must depend on autopsy studies, national cancer registries, and population-based surveillance data to estimate the frequency of [39]. In India, the incidence rate of HCC based on age varies from 0.7 to 7.5 per 100,000 people per year for males and 0.2 to 2.2 per 100,000 people per year for women. In India, the female to male ratio for HCC is 1:4, and the average age group with HCC is 40–70 years [40]. The age-dependent death rate for HCC in India is 6.8/100,000 for males and 5.1/100,000 for women [39]. Cirrhosis is found in 70–97% of HCC cases and is one of the most prominent risk factors that cause HCC globally [41]. The World Gastroenterology Organization lists the following risk factors for HCC: chronic hepatitis B or C, obesity (NAFLD), aflatoxin (cofactor with HBV), tobacco, tyrosinosis, hemochromatosis, alcoholic and primary biliary cirrhosis, a1-antitrypsin deficiency, autoimmune chronic active hepatitis, diabetes, and viral load [42]. It was also noted that males had more chances of developing HCC as compared to females.

**Fig. 3:**
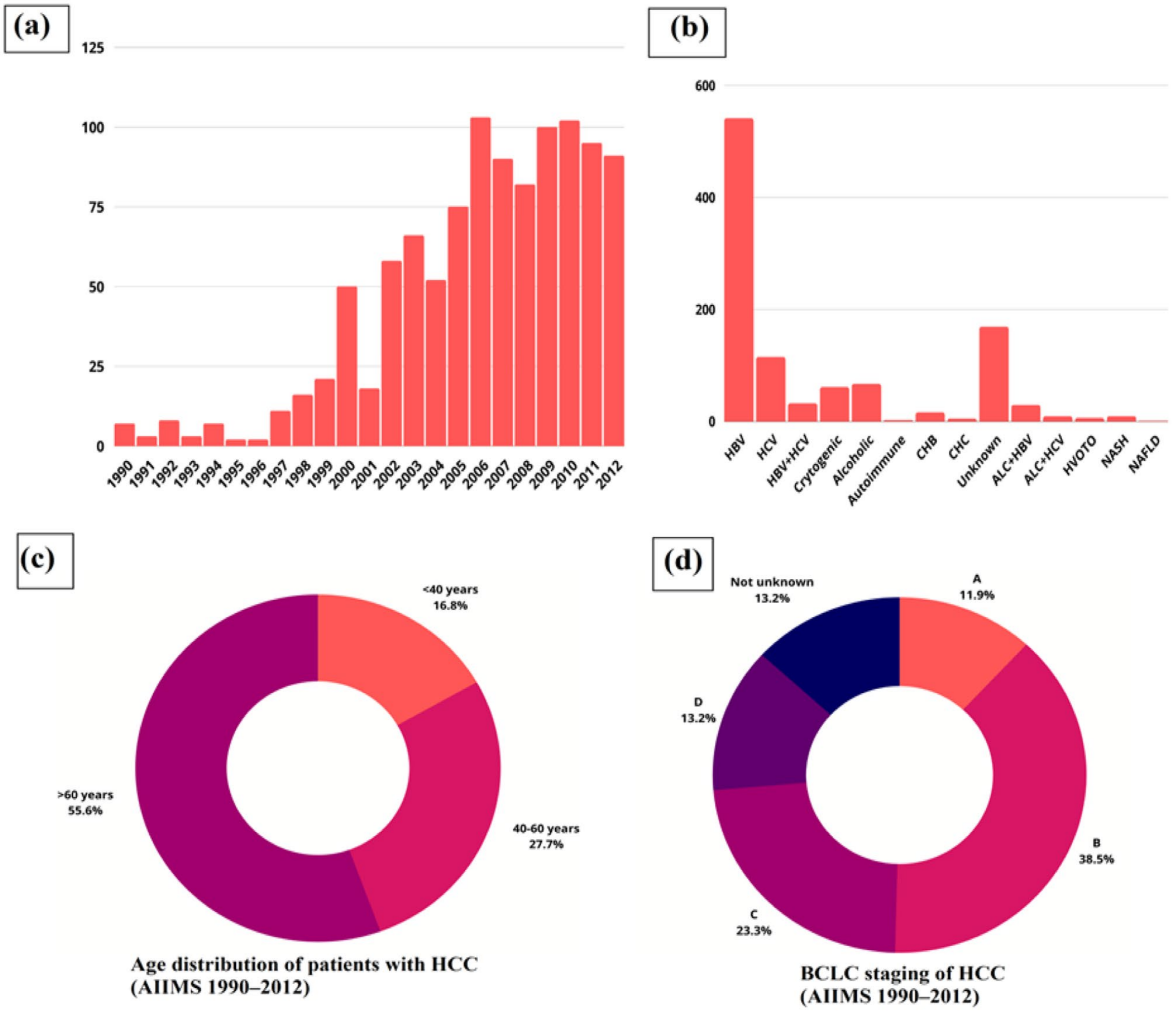
Trends in HCC cases in India from 1990 to 2012. **a** The graph represents the year-wise distribution of patients with HCC in the country. **b** Graphical representation of etiology of HCC in India. **c** Pie chart representing the age distribution of patients with HCC. **d** BCLC staging of HCC. The figure depicts the Barcelona Clinic Liver Cancer (BCLC) staging and indicates that more than 50% of HCC patients of presentation have BCLC-A and B staging, which were offered treatment were as an equal proportion of patients had advanced stage (BCLC-C and D). Data were taken with permission from ref. [40], copyright@2014 (JCEH)

The occurrence of HCC among patients with cirrhosis in India is 1.6% each year during a 563-person-year follow-up period [41]. Furthermore, the incidence of HBV infection in non-tribal and tribal populations was 2.4% and 15.9%, respectively, in a comprehensive analysis of 54 Indian studies [43]. As a result, the yearly number of HCC cases related to HBV is predicted to be between 25 and 35,000. In addition, many HCC patients may be HBeAg-positive, indicating a reduced HBV DNA burden that is linked to a decreased risk of HCC. Unpublished statistics from multiple tertiary health centers in India indicate that the prevalence of HCC is rising. From 1990 to 2012, 1062 individuals with confirmed diagnoses were reported at the All India Institute of Medical Sciences (AIIMS) in New Delhi’s liver clinic. Figure 3 discusses this trend in the number of HCC cases from 1990 to 2012.

Cirrhosis of the liver caused by HBV, HCV, excessive alcoholic intake, exposure to aflatoxin, and NAFLD are significant risk factors that cause HCC in India. According to reports from tertiary healthcare institutions in India, 70–97% of patients with HCC showed liver cirrhosis when diagnosed. On the other hand, reports from Europe and the US found that the yearly occurrence of HCC in HB20, HCV, or alcohol-induced cirrhosis varied from 2.2%, 3.8%, and 1.7%, respectively [44, 45]. Also, the incidence of HCC is more rampant in East Asian nations [46–48]. Although India has roughly 40–45 million HBV carriers and 10 million HCV-infected persons, cancer registries through the Indian Council of Medical Research (ICMR) have reported a reduced prevalence of HCC compared to South East Asia, Japan, and European nations [41].

#### USA

HCC is the most rapidly developing form of cancer in the US, with an increasing incidence and death rate and poor survival [45, 49, 50]. HCV, HBV, alcohol-related liver disease (ALD), and metabolic conditions like non-alcoholic fatty liver disease (NAFLD), obesity, and type-2 diabetes are all significant causes of liver cancer. El-serag et al. [51], Chang et al. [52], Pinheiro et al. [53], and Miller et al. [54] found that these diseases have different profiles depending on the race, gender, and place of birth, which contributes to a considerable variation in a diverse population. It has been noted that chronic HCV infection affects every age group; however, it mainly affects the 1945–1965 birth cohort, which has been a significant driver of increases in incidence and death from liver cancer and HCC in the US [51, 55]. Therefore, the Centers for Disease Control and Prevention (CDC) encourages people to undergo HCV screening for the “baby boomers” cohort in the US since their HCV prevalence is up to five times more than the national average [55].

**Fig. 4:**
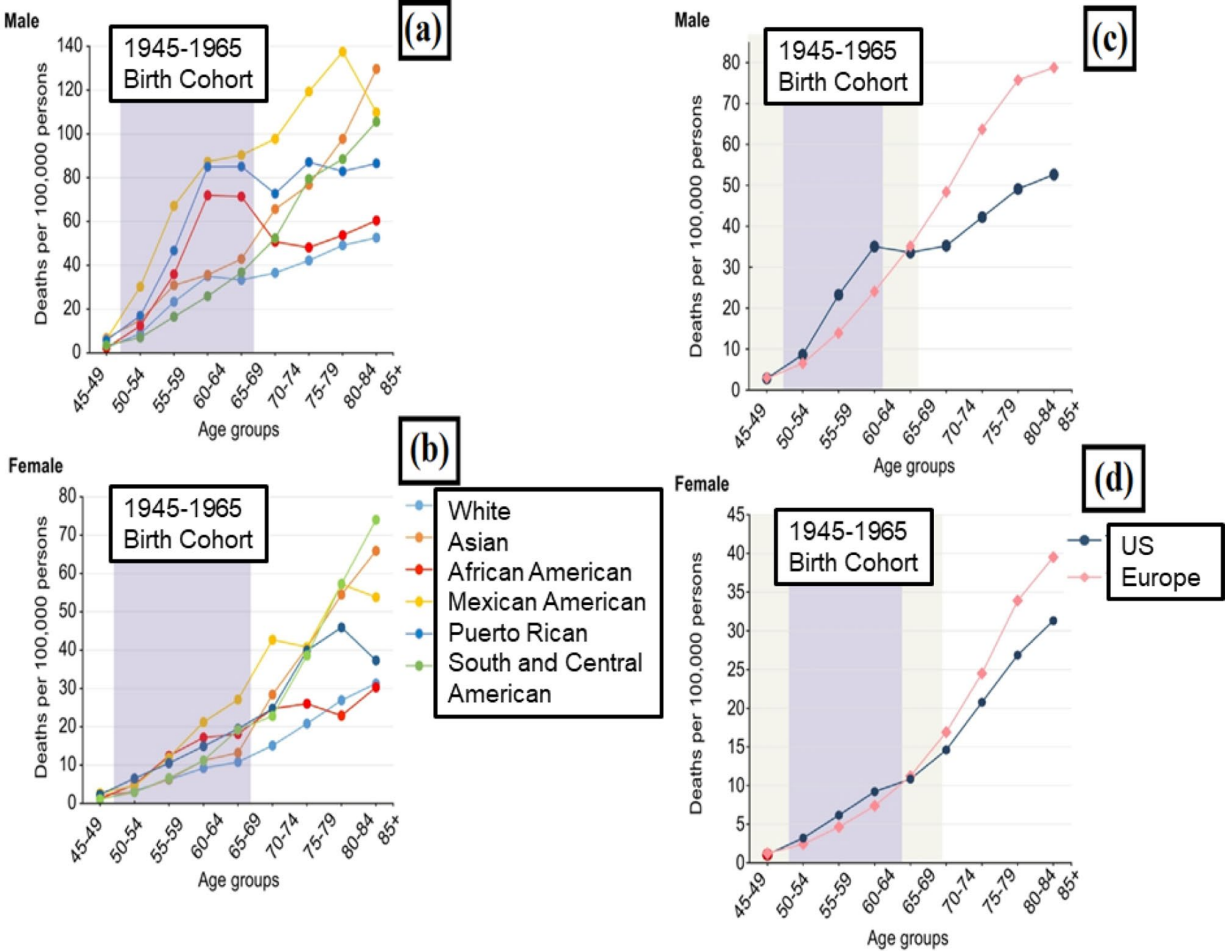
Liver cancer mortality cases across the United States. **a**, **b** Age-specific, sex-stratified liver cancer mortality rates by selected racial/ethnic groups: California, Florida, and New York in 2012–2016. **c**, **d** Age-specific, sex-stratified liver cancer mortality rates, US Whites and Northern and Western Europeans. US Whites include California, Florida, and New York in 2012–2016; Northern and Western Europeans in 2012–2015. Tan regions highlight areas where the 1945–1965 birth cohort overlaps with younger or older cohorts. Reproduced with permission from [57], copyright@2019 (Elsevier)

Compared to other examined demographic categories, including non-Hispanic whites and Asians, Rican and African Americans (US-born), males exhibited a relatively increased age-specific rate (ages 50–69). Surprisingly, this category demonstrated more excellent rates than their senior age equivalents (ages 70–74), providing a confusing “hump and dip” figure rather than the gradual rises in death due to HCC that are generally found as people get older [56]. However, if the typical broad groups of “Hispanic” and “Black” were studied, these significant variations in HCC threat presumably connected with HCV would have been ignored. Death due to liver cancer cases and associated disparities among the older generation are exceptionally substantial in minority populations in previous research [56]. However, in most cases, the surplus is measured concerning Whites, the most influential ethnic group in the US. Therefore, such death rates among the US white senior population can be expected on the other hand. Figure 4 depicts this trend in the number of HCC cases across the US population.

Among males and women, the fraction of the overall mortality rates among the people from the 1945–1965 birth cohorts were most significant in the US-born categories. African American men and women showed the most significant relative liver cancer-based mortality rates, 59% for males and 46% for women, during the 1945–1965 cohort. Except for the South Asian females, all communities with the most foreign-born residents had a relatively modest part in the 1945–1965 generation. Among the 1945–1965 cohorts, the lowest death rates were seen among the South Asian males (24%) and Japanese females (19%). As per the above studies, it was concluded that the mortality rates for liver cancer and HCC have increased with an increase in age. Notably, Mexican immigrants, South/Central Americans, and Asian males have increased death rates with age increases. However, rates decline among African American and Puerto Rican males at an unexpectedly low rate as they become older. In addition, except for African Americans, females' age-specific rates increase with age for all ethnic groupings. Finally, US Whites displayed a constant trend based on age and birth year groupings compared to Northern and Western Europeans. Males in the US had a 49% greater death risk due to liver cancers than men in Northern and Western Europe, between 50 and 64%.

### Gulf Cooperation Council

Countries including Qatar, Oman, Bahrain, Saudi Arabia, and the United Arab Emirates (UAE) make up the Gulf Cooperation Council (GCC). There are conflicting data on the frequency and mortality rates related to HCC in the GCC. However, most research to date has listed HCV as the most prevalent (approximately 45% cause of HCC, followed by HBV infection at 27%) of the cases [58]. The substantial increase in the number of HCC cases, on the other hand, indicates that the cause of HCC may soon shifts from chronic viral liver disease to non-viral liver disease. HBV and HBC infections have been reported to cause 70% of HCC cases detected in GCC. HCV has been found in high incidence in the general population of nations such as Bahrain (1.2%), the UAE (1.3%), Qatar (1.6%), and Egypt (6.3%) [59].

**Fig. 5:**
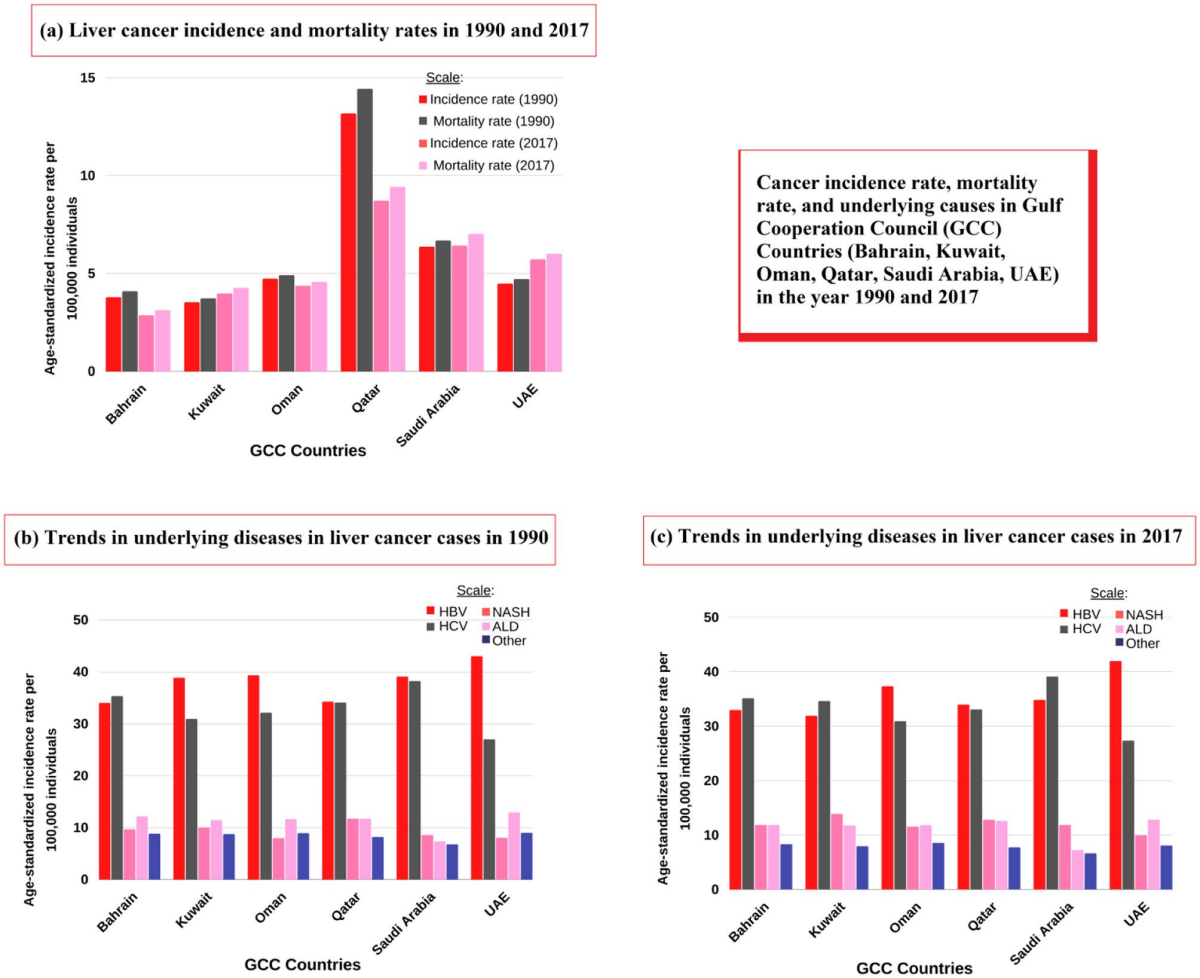
Trends in HCC cases in the GCC in 1990 and 2017. **a** The graph represents the incidence and mortality rates of liver cancers in 1990 and 2017. **b** Graphical representation of underlying liver cancer disease conditions (HBV, HCV, NASH, ALD, others) in 1990 across the GCC. **c** Graphical representation of underlying liver cancer disease conditions (HBV, HCV, NASH, ALD, others) in 2017 across the GCC. Data were taken with permission from [62], copyright@2020 (Wiley)

Between 1990 and 2017, the incidence rate of HCV-related HCC rose by 16.1%. Egypt and Qatar are dominant countries that plan to eliminate HCV by 2030 [60, 61]. From 1990 to 2017, the ASIR for liver cancer grew by 11.91%. Between 1990 and 2017, the ASIR of liver cancer rose in the UAE (+ 27.68%), Kuwait (+ 12.46%), Qatar (− 33.79%), Oman (− 7.61%), Saudi Arabia (+ 0.94%), and Bahrain (− 24.34%). Figure 5 discusses this trend in the number of cases across the GCC.

## Selected Plants and Their Anticancer Activity Against Hepatocellular Carcinoma

**Fig. 6:**
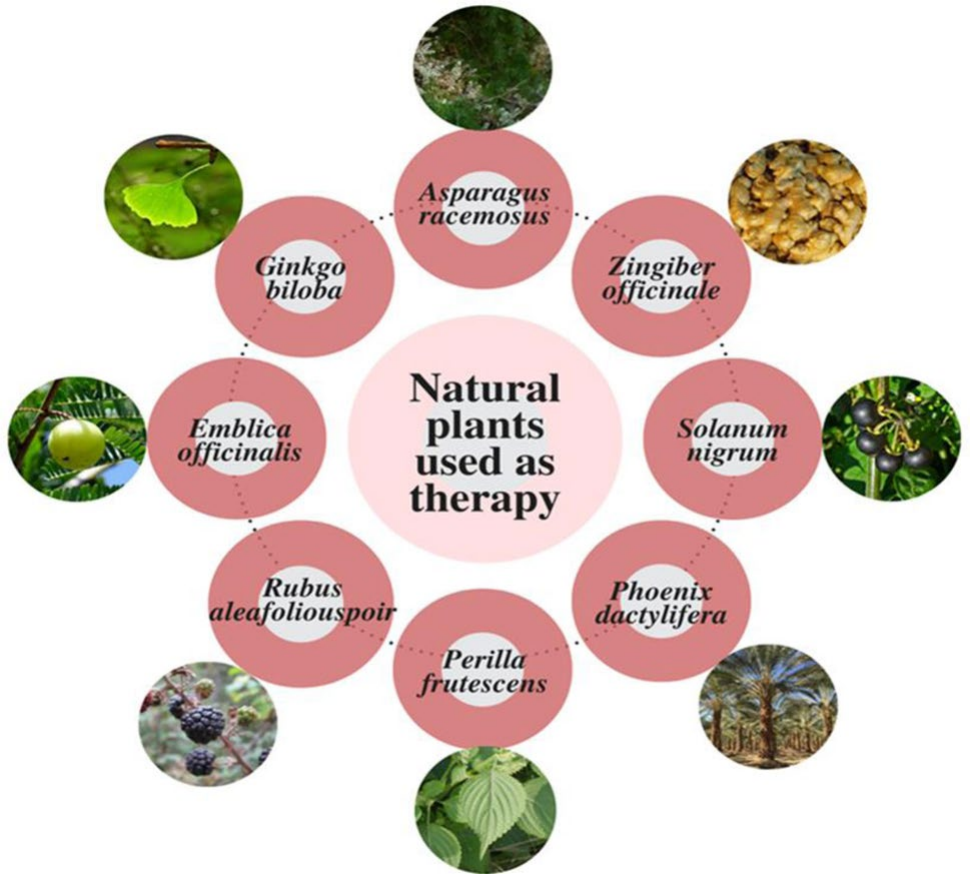
Plants used as a therapy to treat advanced cirrhosis stage and liver cancers

Natural herbal medicines have been shown to possess several health benefits [63–66]. Many natural plants have been shown to possess the ability to treat liver cancers and cirrhosis; these are mentioned in Fig. 6.

### Asparagus Racemosus

The genus *Asparagus*, widespread in India, Asia, Australia, and Africa, includes 300 species globally [67]. Historical sources show the use of plants from the genus over centuries in classical medicine. *Asparagus racemosus* is a perennial plant, which grows as tall as 7 m (23 ft) and belongs to the family *Asparagaceae*, which possess a stiff stem, needle-like leaves, and small white flowers. Shatavari (*A. racemosus*) is a well-known Ayurvedic Rasayana that helps to slow down the aging process and boost immunity, neuropathy, and hepatopathy [68]. It is also used for the preparation of Chyawanprash [69–71]. Various studies have revealed that aqueous root extracts of *A. racemosus* exhibit anticancer activity, anti-inflammatory, antioxidant, and immunomodulatory properties [72].

Extractions are derived from dried roots of *A. racemosus*. The main pharmacological compounds synthesized by *A. racemosus* include steroidal saponins, essential oils, various amino acids, flavonoids, resin, steroidal tannin glycosides (asparaguses), and bitter glycosides. Other primary chemical constituents reported from roots and leaves of *A. racemosus* include diosgenin and shatavarins I and IV [73]. These saponin glycosides derived from the roots of *A. racemosus* can prevent hepatocellular carcinoma experimentally induced by diethylnitrosamine (DEN). When the hepatic tissues of DEN-treated rats are stained immunohistochemically, clusters of cells with mutated p53 antigen are observed. As per this study by Agrawal and colleagues, Wistar rats were given an aqueous extract of racemosus roots, showed no case of hepatocarcinogenesis. Furthermore, DMBA, the induced mammary tumor, showed a sharp decline in rats exposed to *A. racemosus*. Rats fed with a 2% *A. racemosus* diet showed decreased tumor cases and the average number of tumors per tumor-bearing specimens, thus signifying the importance of *A. racemosus* in the treatment HCC [74].

### Solanum Nigrum

*Solanum nigrum* (black nightshade) comes from the family of *Solanaceae* and is distributed throughout the world, and is available in various forms [75]. The *S. nigrum* is native to Eurasian and does not occur naturally in South America [76]. Nevertheless, the plant is frequently used as an essential ingredient to treat pneumonia, stomach ulcers, fever, aching tooth, inflammation, etc. According to recent research, the aqueous extract of *S. nigrum* (AESN) is an essential ingredient in typical Chinese medicine formulations to treat patients with different cancers [77]. The *S. nigrum* has antitumor effects against human melanoma, colorectal, endometrial, breast, and liver cancers [78–81]. In addition, *S. nigrum* exhibits antiproliferative properties in various cancer cells [82, 83], suppressing tumor cell growth primarily through apoptosis induction. *Solanum nigrum* is rich in secondary metabolites such as alkaloids and steroid saponins glycoprotein that exhibit anticancer activity [84, 85]. Recent research has discovered four novel steroidal glycosides, alkaloids solamargine, solasonine, alpha, and beta solanigrinechez, inhibit cell proliferation and cause apoptosis in uncontrolled cancers [86]. Water extracts of *S. Nigrum* protect rats from CCl_4_-induced chronic hepatotoxicity [87]. By modulating the antioxidative defense mechanism, *S. nigrum* can minimize CCl_4_-induced lipid peroxidation. Elshater et al. discovered that rats given *S. nigrum* after 30 days after the CCl_4_ challenge showed a substantial decrease in liver marker enzymes, lipid peroxidation, and an increase in enzymatic and non-enzymatic antioxidative defense mechanism [88].

### Rubus Aleafolious Poir

*Rubus aleaefolius*, commonly called Elm-Leaf Blackberry, belongs to a family of *Rosaceae* and is native to Europe, Africa, and is also naturalized in Kashmir. The *R. aleaefolius* is a folk medicine used to cure a variety of hepatic diseases, including HCC. The *R. aleaefolius* contains numerous essential medicinal chemicals, including butanol and ethyl acetate, has shown hepatoprotective properties in mice with acute liver damage following exposure to CCl_4_, as per the study carried out by Hong et al. Zhao et al. scrutinize extractions of total alkaloids from *R. aleaefolius* (TARAP), used as an antimetastasis medication to treat HCC in both in vitro and in vivo conditions.

HCC development has shown to be influenced by TARAP with the induction of apoptosis via the caspase 3 and 9 pathways in HepG2 cells and by mitochondrial-mediated apoptosis [89]. Many human cancers, including HCC, have constitutively activated signal transducer and activator of transcription 3 (STAT3) pathway, which plays an essential role in cell proliferation and multiplication in such cancers. This STAT3 phosphorylation can be suppressed in tumor tissues by the TARAP treatment [90]. Furthermore, TARAP has been shown to change the expression of several primary STAT3 signaling pathway target genes, like cyclin D1, cyclin E, cyclin-dependent kinase (CDK) 4, and CDK2 as up-regulating p21. These findings confirm that one of TARAP’s anticancer activity mechanisms against HCC is inhibiting the STAT3 signaling pathway, leading to cell multiplication block and thus arresting the cell cycle.

### Perilla Frutescens

The annual herb *Perilla frutescens* is commonly known as Korean perilla or Beefsteak vine. Perilla’s origins can be traced back to China, Japan, Korea, Taiwan, and Vietnam. This herb is a member of the mint family, *Lamiaceae*, and can be found across Asia and is assessed in its contribution to culinary and traditional medicinal uses. Extractions from this plant’s seed, leaf, and stem parts are used for many therapeutic applications such as poisoning, bloating, cold, and headaches [91]. In addition, *P.a frutescens* has been demonstrated to possess anticancer and antitumor efficacy both in vivo and in vitro conditions.

The *P. frutescens* leaf extract had the best anticancer efficacy in Hep G2 cells, inhibiting cell growth and increasing apoptosis-related gene expression [92]. In addition, Lin et al. used leaf extracts of *P. frutescens* to evaluate its inhibitory effects in human hepatoma Hep G2 cells. They discovered that it efficiently activates apoptosis-related genes and inhibits cell growth. Other investigations found that an ethanolic leaf extract of *P. frutescens* increased apoptosis and tumor formation by combining death-receptor-mediated apoptosis with the scavenging of reactive oxygen species (ROS) [93, 94].

### Ajwa Dates (*Phoenix dactylifera* L.)

The Al-Madinah Al Munawara and its surrounding areas in Saudi Arabia are home to the most common natural fruits, mainly date fruit (*Phoenix dactylifera* L.) [95]. The anticholesteremic, antidiabetic, anti-inflammatory, antioxidant, hepatoprotective, and anticancer properties of the Ajwa date have been identified in conventional and alternative medicines [96, 97]. Phytochemicals in Ajwa fruits (flavonoids glycosides, polyphenol, and phytosterols) [98, 99] exhibit anti-inflammatory, antioxidant, cardioprotective, and antiapoptotic properties [100]. An aqueous extract of Ajwa dates was found to reduce and block diethylnitrosamine-induced liver carcinoma in a rat model [101]. Pulp also includes phenolics, including quercetin and kaempferol [99], which have been shown to have anticancer activity against HCC cells, according to other reports [102, 103].

## Anticancer Activity of Extracts and Compounds from *Curcuma longa* and Its Treatment for Hepatocellular Carcinoma

In recent years, there has been a revival of interest in studying the usage and effectiveness of medicinal herbs to treat various diseases. In this section, we have performed a deep analysis of the properties of *Curcuma longa* for the treatment of liver cancers, particularly HCC, and have also discussed its mechanism of action in detail. Curcumin, with the scientific name “*Curcuma longa*,” the chemical name “diferuloylmethane,” and the chemical formula C_21_H_20_O_6_, is an essential ingredient of turmeric plants.

### Chemical Structure of Curcumin

Curcumin, a yellow pigment from *C. longa*, makes up 2–8% of turmeric compounds and is generally used as a food coloring agent and spice. The *C. longa* belongs to the *Zingiberaceae* plant family and is widely distributed in south-eastern and southern tropical Asia [104]. Rhizomes are the most commonly used plant component. They contain many compounds, including bioactive non-volatile curcuminoids like curcumin, dimethoxy, bisdemethoxycurcumin, and volatile oil compounds like sesquiterpenoids [105, 106]. Due to its hydrophobic nature, curcumin is almost insoluble in a neutral solvent (i.e., water) and readily soluble in organic solvents, and possess low inherent toxicity and properties including antioxidant, anti-inflammatory, antimicrobial, antitumor, antidiabetic, hypocholesterolemic, antithrombotic, antihepatotoxic, antidiarrheal, carminative, diuretic, antirheumatic, hypotensive, antioxidant, larvicidal, insecticidal, antivenomous, and antityrosinase effects [107–110]. Curcumin’s anticancer activity is recognized as one of its most important effects, owing to its low cytotoxicity to normal cells. Antiproliferative effects have also been observed in various cancer cell lines, including prostate, breast, colorectal, pancreatic, and kidney cancers [111]. When consumed as 0.1–3 mg/kg body weight, curcumin has been shown to inhibit the telomerase reverse transcriptase enzyme and lower Bcl-2 expression [112, 113].

### Mechanism of Action of Curcumin

**Fig. 7:**
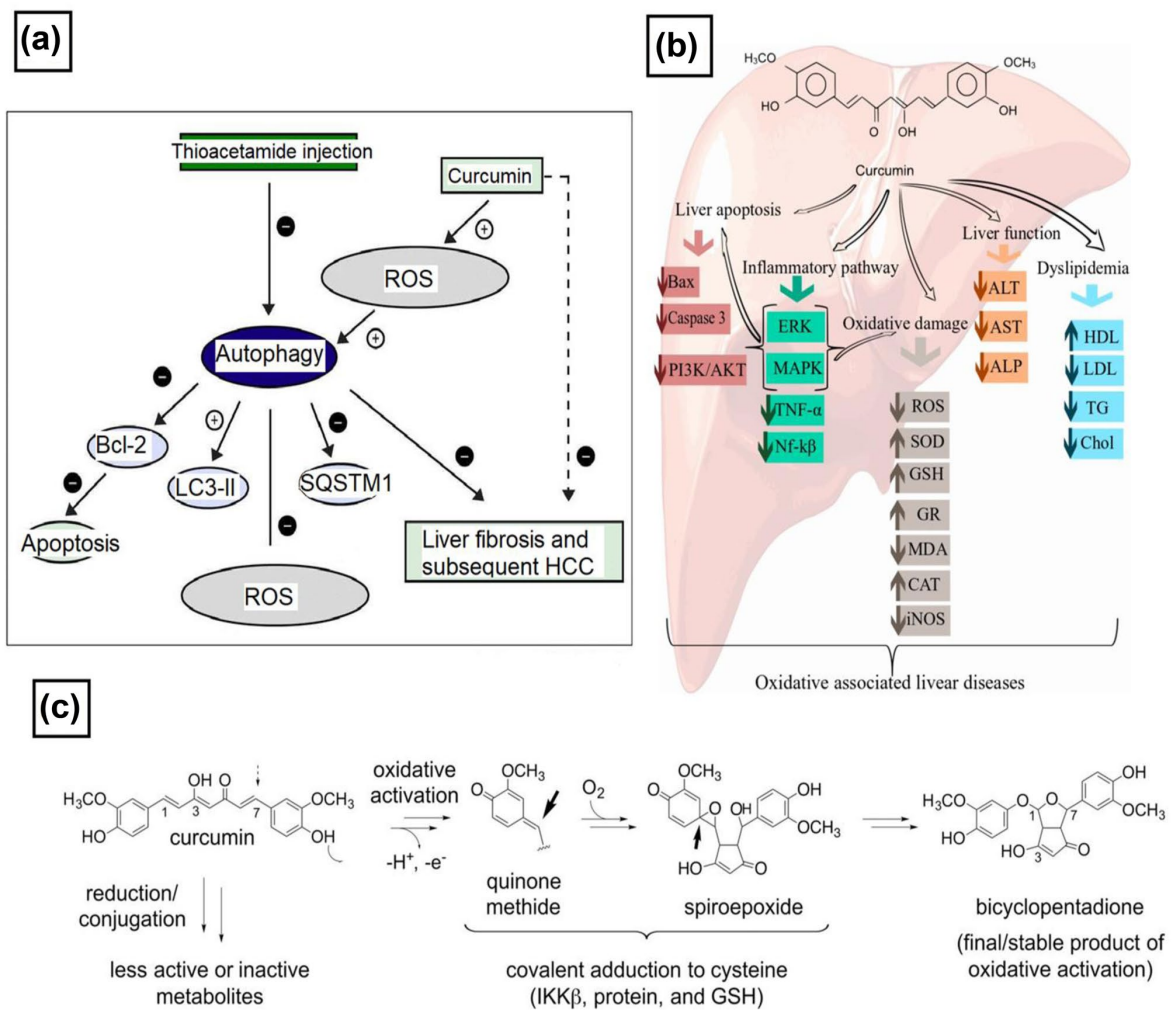
Mechanism of curcumin action in the treatment of HCC. **a** The postulated mechanism for the effect of curcumin on autophagic and apoptotic pathways and ROS in thioacetamide-induced HCC. Adapted with permission from [118], copyright@2017 (Elsevier). **b** Cellular and molecular mechanisms of curcumin in the prevention of oxidative-associated liver disease. Alanine aminotransferase (ALT), aspartate aminotransferase (AST), alkaline phosphatase (ALP), ROS, sulfasalazine reduces superoxide dismutase (SOD), glutathione (GSH), glutathione reductase (GR), malondialdehyde (MDA), catalase (CAT), inducible nitric oxide (iNOS), high-density lipoprotein (HDL), low-density lipoprotein (LDL), triglyceride (TG), extracellular signal-regulated kinases (ERK), and mitogen-activated protein kinase (MAPK). Adapted with permission from [119], copyright@2018 (MDPI). **c** Biological activities of curcumin. **c** Mechanism of oxidative bioactivation of curcumin, initiated by hydrogen abstraction from a phenolic hydroxyl of curcumin. The resulting quinone methide radical forms a cyclopentadiene ring and adds molecular oxygen to give a spiroepoxide intermediate that undergoes further transformation to the final dicyclopentadiene. Adapted with permission from [120], copyright@2017 (JBC)

Curcumin has been shown to inhibit carcinogenesis by influencing angiogenesis and cancer cell development. It also indicates the antiangiogenesis effect by inhibiting angiogenic factor stimulators, including VEGF and primary fibroblast growth factor. Curcumin inhibits IL-8 expression by blocking VEGF expression through NF-kB, AP-1 regulation [114], and the PI3K/Akt signaling pathway [115]. Curcumin also suppresses angiogenic cytokines, including IL-6, 23, and 1, which inhibit angiogenesis in certain tumors. It may also cause cancer cells to die by inducing apoptosis via a p53-dependent pathway. Several studies have shown that curcuminoid compounds serve as free-radical scavengers by minimizing lipid peroxidation caused by free radicals [116]. Curcumin-mediated suppression of nuclear factor B (NF-B) allows regulating the inflammatory cascade in most chronic illnesses, including cancers [117]. This can be better understood from Fig. 7 that describes the mechanism of curcumin action in HCC treatment.

Various studies have shown that curcumin has a high potential for treating various inflammatory diseases [120–122] and can (i) block pro-inflammatory transcription factors (NF-kB and AP-1); (ii) decrease pro-inflammatory cytokines TNFα, IL-1b, IL-2, IL-6, IL-8, MIP-1a, MCP-1, CRP, and PGE2; (iii) down-regulate enzymes such as 5-lipoxygenase and COX-2 and COX-5; and (iv) inhibit MAPK and pathways involved in nitric oxide synthase (NOS) enzymes synthesis [121, 123–125].

### Curcumin Stimulates Apoptosis of Liver Cancer Cells

**Fig. 8:**
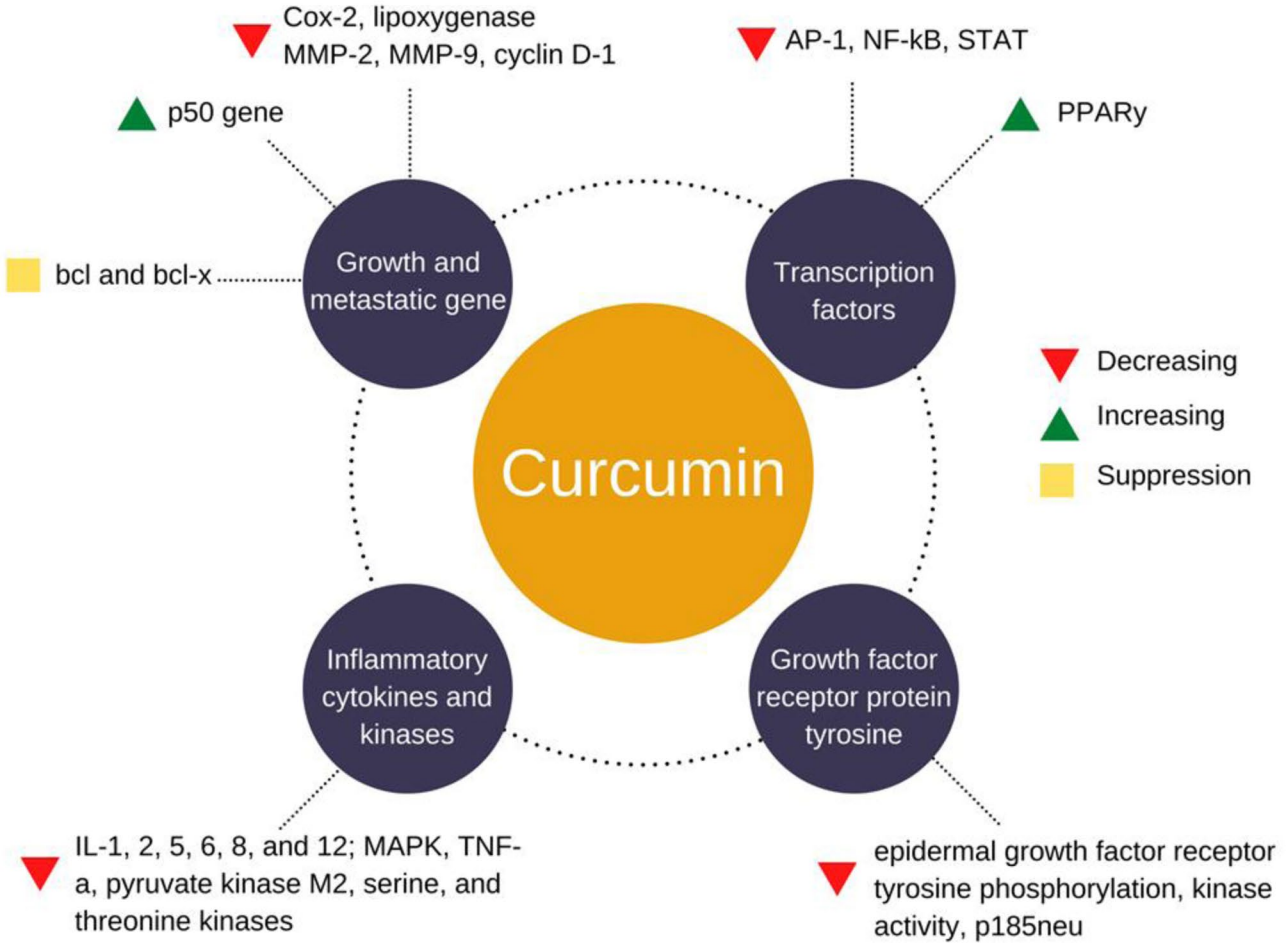
Molecular targets of curcumin when used against liver cancers. Reused with permission after changing the design from [130], copyright@2020 (Frontiers)

Curcumin can stimulate apoptosis of liver cancer cells [126]. Extrinsic and intrinsic mechanisms of action of curcumin can both induce programmed cell death. Internal stimuli such as DNA abnormalities, ischemia, viral infection, and cellular distress activate the intrinsic route [126]. Death receptors from the TNF receptor gene superfamily are involved in the extrinsic (receptor-mediated) pathway [127]. According to a study by Liu et al. EF24, a synthetic molecule and a robust curcumin analog with increased bioavailability can effectively decrease HCC and promoted apoptosis in a mouse liver cancer cell line [128]. Compared to control (non-EF24-treated) groups, authors noted that cytochrome c, cleaved-PARP, Bax, and activated caspase-3 levels were high, whereas PARP and Bcl-2 were down. In addition, curcumin treatment of human hepatoma SMMC-7721 cells for 24 h can lower BCL-2 protein expression [129]. Another study on mouse liver cancer cells observed that EF24 causes cell cycle arrest in the G2/M phase. The activation of CDC-2 by cyclin B1 is required for the transition from G2 to M-phase. The authors recorded that the cells’ levels of cyclin B1 and CDC-2 were dramatically lowered when curcumin was used [128]. In addition, curcumin administration prompted the activation of the Chk1-mediated G2 checkpoint, resulting in G2/M arrest and resistance of malignant cells to curcumin-induced apoptosis, as shown in Fig. 8.

In another study by Wang and coworkers also revealed the apoptotic pathway of curcumin in treating liver cancers. In addition, the authors demonstrated that the apoptosis-inducing effect of curcumin is linked with mitochondrial apoptosis. Herein, upon the secretion of cytochrome c, caspase-9 gets activated, which cleaves caspase-3 and polymerase 1, which then paves the path to cell death [131].

### Curcumin Prevents Metastasis and Tumor Progression

**Fig. 9:**
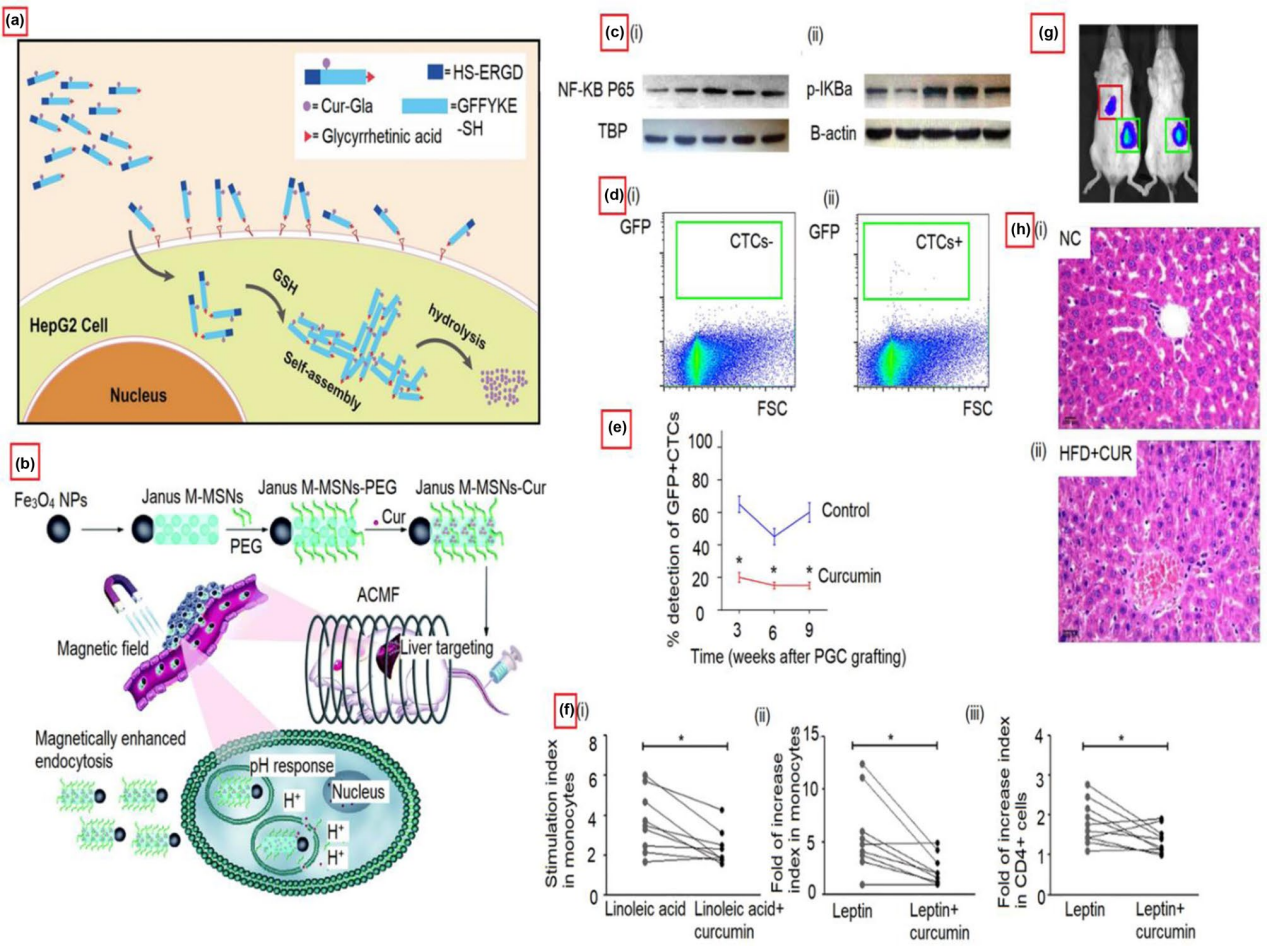
Curcumin action in treating liver cancers. **a** Illustration of the mechanism of curcumin compound (GA–GFFYK(Cur)E-ss-ERGD) for tumor targeting therapy. The compounds were created by modifying curcumin supramolecular pro-gelator (GA-Cur) with glycyrrhetinic acid (GA) and a compound Nap-Cur after displacing GA with the naphthylacetic acid (Nap). Adapted with permission from [133], copyright@2017 (Nature). **b** Schematic diagram showing the action of Janus magnetic mesoporous silica nanocarriers for magnetically targeted and hyperthermia-enhanced curcumin therapy of liver cancer. Adapted with permission from ref. [134], copyright@2018 (RSC). **c** (i and ii) Effects of curcumin on the hepatic GRP78 protein expression and the ratio of p-PERK/PERK and-IRE1α/IRE1α in alcohol-induced liver injury. Adapted with permission from [135], copyright@2019 (Food & Nutrition Research). **d** (i and ii) Representative flowcharts for a negative detection or a positive detection of circulating tumor cells by flow cytometry showing the reduction of stem cells (s.c.) grafted primary gastric cancer cells (PGCs) in the presence of curcumin. **e** Curcumin reduces circulating tumor cells (CTCs) of s.c. They grafted PGCs. The ratio of detection of CTCs. *p < 0.05. *N* = 30. Adapted with permission from [136], copyright@2019 (Aging). **f** The reversal effects of curcumin on peripheral immunological cells. Ex vivo curcumin treatment of peripheral blood mononuclear cells from patients with NAFLD resulted in decreases in (i) linoleic acid-induced ROS generation and (ii) leptin-induced TNF-α production by monocytes. (iii) Ex vivo curcumin treatment of peripheral blood mononuclear cells from patients with NAFLD resulted in decreased IFN-γ production in CD4 + cells. Lines connect the “linoleic acid” and “linoleic acid + curcumin” stimulation indexes or the “leptin” and “leptin + curcumin” fold of increase indexes for each patient. Adapted with permission from [137], copyright@2017 (PLOS ONE). **g** Curcumin reduces metastatic tumor formation in the liver of s.c. Grafted pooled primary gastric cancer cells that also affected the hepatic cells. Representative images show a positive and a negative case by bioluminescence in the liver area. Adapted with permission from ref. [136], copyright@2019 (Aging). **h** Effects of curcumin (CUR) on a high-fat diet (HFD)-induced hepatic steatosis. NC: Normal cells. Adapted with permission from ref. [137], copyright@2021 (Frontiers)

TNF-α has a critical function in tumor cell survival and malignancy. This TNF-α expression can be inhibited by curcumin. However, curcumin's hydrophobicity and limited bioavailability can become substantial roadblocks. To overcome this, curcumin can be encapsulated in microcells to create a sustained release formulation and thus improve its solubility and bioavailability [132]. Compared to the free form of curcumin, such curcumin-bearing microcells can dramatically lower the levels of liver enzymes in the HCC-induced animal groups. In addition, curcumin-containing microcells can also stimulate the production of pro-apoptotic molecules such as p53 and Bax. Figure 9 explains the curcumin action in tumor progression prevention and metastasis.

#### Curcumin’s Effect on the Biochemical Profile

Choudhury et al*.* found that a curcumin injection (8.98 M) lowered NADH oxidase and elevated GR, GST, and succinate dehydrogenase activity in Swiss albino rats with CCl_4_-induced hepatotoxicity [138]. Curcumin treatment (200 mg/kg) in Sprague–Dawley rats enhanced hepatic glutathione levels. It lowered lipid peroxidase levels and the activities of both ALT and aspartate AST for the same kind of hepatotoxicity [139]. Curcumin may thus be a potential drug for preventing oxidative stress-related liver disease by lowering ALT, AST, and alkaline phosphate levels, raising GST, GR, GPx, SOD, and CAT levels, and reducing NO and suppressing ROS generation [119]. In addition, curcumin therapy can boost endogenous antioxidant levels (ascorbic acid, GSH, SOD, and CAT) in the liver of chronic iron overloaded male rats, according to Badria et al. [140].

### Positive and Negative Side Effects of Using Curcumin to Treat Hepatocellular Carcinoma

With the recent advances in science and technology, several options serve as therapeutic options to treat hepatocarcinoma [141]. Pathogenesis of hepatocarcinoma is a multi-step process, and the extent of angiogenesis determines the therapeutic option to be used. Most common include surgery, radiofrequency ablation, embolization, and liver transplant. Immunotherapeutic prospects are also promising to treat melanomas; the treatment option used to treat HCC depends on the cancer stage. Recently, trans-arterial chemoembolization (TACE) has been combined with sorafenib to block neovascularization during tumour development and proliferation [142]. Though these methods are often successful, conditions like patient’s response and performance and liver function are also crucial, determining factors for HCC treatment.

Further research needs to be done to avoid uncertainties and side effects these treatments follow. For instance, though TACE in combination with sorafenib has shown positive results, it is still uncertain to recommend their exact dosage, frequency, and duration during the entire treatment. In addition, recent studies have deemed the newer modalities ineffective and do not provide any therapeutic benefit during the treatment of HCC [143]. Also, with the increasing medical expenses, it’s difficult for all patients to afford the prescribed option for targeted therapy. High death rates hinder most of the available HCC treatment options due to the recurrence of cancer or resistance of tumours against the therapeutic option used [144]. Options like radiation therapies are minimal in terms of the advanced prognosis of the tumour. Radiotherapy does not allow for precise location of the tumour margins and often destroys the normal cells in the patients. Therefore, alternative treatment options are necessary to increase their response rate and lower the toxicity resulting from the treatment. This is why curcumin serves as a promising therapeutic option for HCC treatment.

The significant advantage of curcumin as a chemotherapeutic agent is its ability to be used alone or in combination with other potential chemotherapeutics without showing any adverse effects like neurotoxicity or peripheral neuropathy [145]. Furthermore, its anti-inflammatory, hepatoprotective, antimutagenic properties, curcumin also helps combat issues like gastrointestinal inflammation that arise due to chemotherapies or radiotherapies [146]. Post chemotherapy, patients are prone to multiple infections. Curcumin, possessing anti-infective properties, easily helps overcome major and minor ailments [147]. Other positive side effects of curcumin include its effect on cell-cycle progression, invasion, epithelial-mesenchymal transition, and drug resistance shown by cancer cells [148]. Furthermore, Curcumin has been shown to perform immunoregulation of HCC by monitoring the miR-21/TIMP3 axis [149]. Shao et al. demonstrated that curcumin suppresses tumour proliferation by down-regulating lincROR and inactivating the Wnt/β-catenin signalling, thus placing a crucial anticancer role during the treatment of HCC [150]. In another study by Bose and colleagues, the authors concluded that curcumin affected the ratio of CD4 + T cells to CD8 + T cells by lowering the regulatory T-cells and reducing the T-cell apoptosis and thus enhancing the immune response against cancer cells in HCC [151].

A recent study by Tian and colleagues showed that when used along with drugs like paclitaxel, curcumin enhances the body’s response towards HCC treatment and reduces chemoresistance [152]. In another similar study, curcumin, piperine, and taurine, when used in combination, affect interleukin-10 and miR-21 levels and, therefore, increase anticancer activity in HCC patients [153]. Likewise, in another study by Man and coworkers, it was concluded that the addition of curcumin in diet strengthened the overall antitumor efficacy in the body and overcame most limitations posed by current treatment options like sorafenib [154]. Thus, curcumin can be combined with other existent options to treat hepatocellular carcinoma better.

In addition, curcumin also suppresses the activity of pro-angiogenic proteins, like vascular endothelial growth factor [23], COX-2, and basic fibroblast growth factors; inhibits cell motility, cellular adhesion molecules, endothelial cell migration, metastasis and related complications, and extracellular proteolysis [155]. Furthermore, curcumin displays antiproliferative, low immunogenicity, and proapoptotic effects on cancer cells [149]. These exceptional properties of curcumin, in addition to no toxic effects, show promising potential for curcumin to be used in combination with other techniques for the treatment of hepatocellular cancer.

Generally, curcumin is considered safe for moderate consumption and is commonly used in South Asian households. However, few cases of colorectal carcinoma have been reported in association with curcumin when consumed in large doses [156]. When ingested in raw form and large quantities, curcumin may lead to gastric irritation, indigestion, diarrhea, allergies, and antithrombosis activity [157]. Apart from its antioxidative properties, curcumin also shows pro-oxidative properties [158]. Based on its concentration, curcumin may either function as an antioxidative agent or as a pro-oxidative agent. Therefore further research must be done to understand better how curcumin functions under changing conditions.

## Risk Factors of Hepatocellular Carcinoma

### HBV

Hepatitis B virus (HBV) is thought to be the most potent epidemiological factor linked to HCC. Nearly half of all HCC cases are caused by chronic hepatitis B (CHB). However, the significance of risk factors varies significantly by area (e.g., more in East Asia but lower in Europe) [159]. More than 250 million people worldwide are affected with chronic HBV or have been exposed to it. Infants are particularly more at risk. About 90% of children infected with HBV become chronic HBV carriers [160]. The rate of HBV carriers is nearly 8% in high endemic regions [161]. In high-incidence areas, 80% of HCC patients show the Hepatitis B surface antigen (HBsAg) [162]. In untreated CHBV patients, the 5-year average incidence of cirrhosis is 8–20%, with a 2–5% annual chance of HCC in people with cirrhosis. Every year, nearly 900,000 people die from HCC caused by HBV. According to WHO, post-COVID-19 era, there will be an additional 5.3 million chronic HBV infections in children born between 2020 and 2030 and an estimated a million deaths due to HBV among them. In addition, the number of children below five years with chronic HBV infection has reduced from 5% in the pre-vaccine period to just under 1% in 2019 [163]. Socio-demographic factors like gender, age, family history, viral etiology, environmental exposure (aflatoxin intoxication, tobacco, alcohol), and dietary factors are linked to higher HCC affliction chances [164, 165]. HBV-driven HCC can develop either via direct or indirect mechanisms, which includes the following: (1) ongoing inflammatory processes that attempt at clearing the disease condition; (2) changes in host genome structures that incorporate HBV DNA sequences and changes in epigenetics; and (3) accumulation of altered forms of HBV envelope proteins and continuous expression of viral proteins with oncogenic potentials like the regulatory HBX protein.

### HCV

**Fig. 10:**
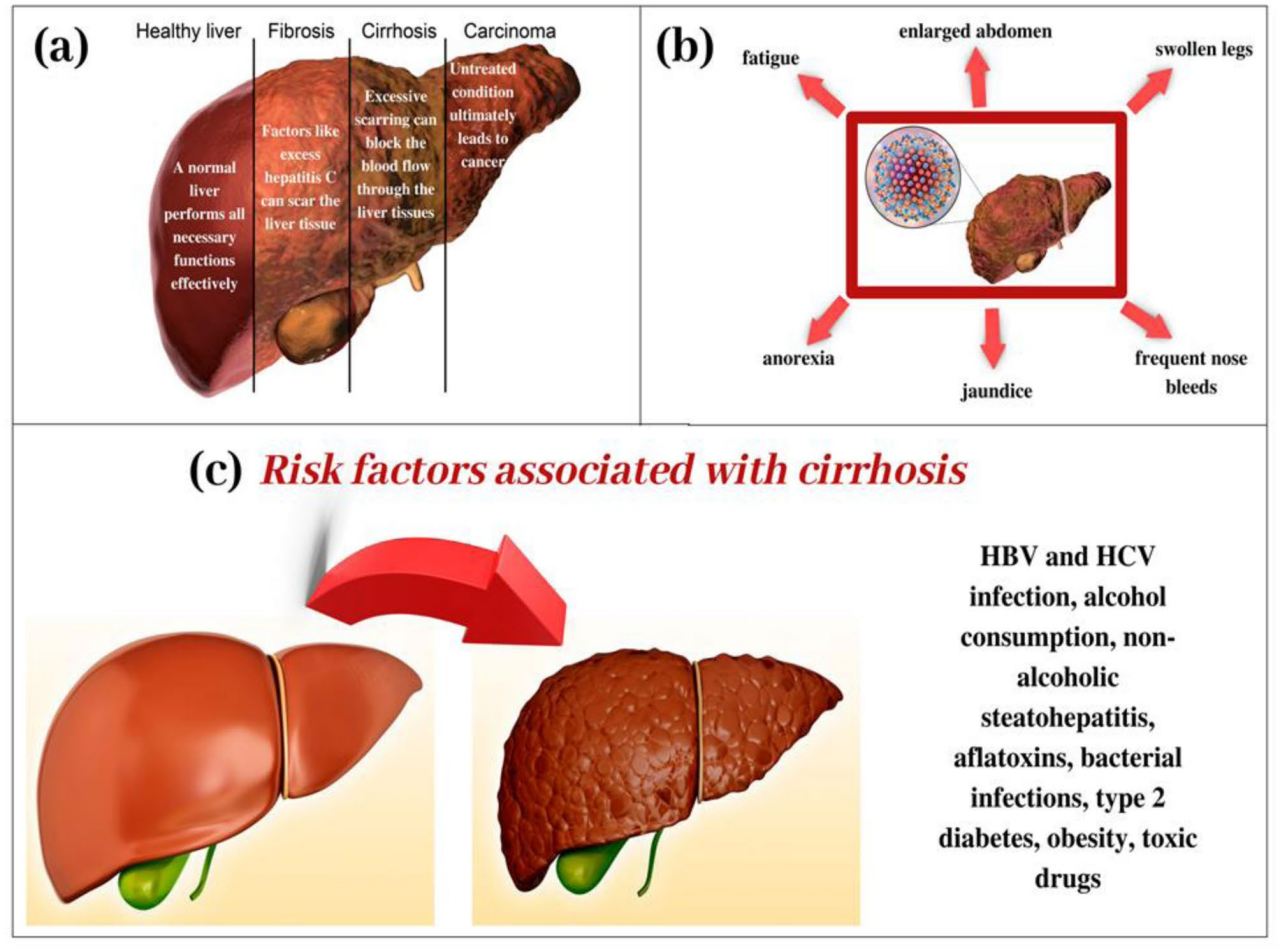
Cirrhosis of the liver. **a** Stages of liver degradation and the associated risks with each stage. **b** Symptoms of liver cirrhosis. **c** Risk factors that cause cirrhosis

Hepatitis C virus (HCV) is a hepatotropic positive single-stranded RNA virus that causes HCC along with other risk factors. HCV-related HCC has the maximum death rates per 100,000 people in US [166, 167]. HCV etiology is found in 30% of Asian HCC patients and 50% Caucasian HCC patients. During 2009–2018 the number and rate of newly reported chronic HCV cases have rapidly increased among young people, with the highest new infections among 20–29 years of age. As per CDC analysis, cirrhosis is most prevalent in patients with HCV-related HCC being approximately 80–90% [168, 169]. In addition, meta-analysis studies showed that the patients with HCV genotype 3 have a 50% increased rate of fibrosis than other genotypes [170]. The risk factors associated with cirrhosis are explained through pictorial representation in Fig. 10.

In cirrhotic cases, HCC occurs more often than in people with mild fibrosis. Mutations cause HCV-HCC in hepatocytes in the context of cirrhosis. HCV proteins are also connected with cell division, metastasis, tumor development, and transformation. When over-expressed, HCV core proteins NS3 and NS5A block tumor suppressor genes TP53, TP73, and RB1 and inhibit negative cell cycle regulators like CDKN1 [171].

### NAFLD/ NASH

Nonalcoholic fatty liver disease (NAFLD)/nonalcoholic steatohepatitis (NASH) is a benign (or recurrent) form of the disease where, histologically, accumulation of fat occurs (steatosis) in > 5% of the hepatocytes [172, 173]. It is anticipated to become the most prominent reason for end-stage liver disease and HCC [2, 3]. Shortly, the occurrence of NAFLD will be simultaneous with an increase in obesity and the event of metabolic syndrome. Such a combination will represent a severe health hazard, causing an increase in liver-related morbidity and mortality, especially in HCC conditions [174–177]. The NAFLD/NASH-related mortality post-liver cirrhosis has risen significantly in the last ten years. HCC in individuals with NAFLD/NASH is also detected in people devoid of cirrhotic conditions. This causes a delayed diagnosis and a worsened tumor burden.

Steatosis alone does not cause HCC since the rampant inflammatory condition is needed for carcinogenesis [178]. The NASH and HCC are the product of several concurrently acting factors, such as genetic changes, impaired lipid metabolism, and insulin resistance [177, 179, 180]. HCC production is aided by hepatocyte cell death, compensatory division, and increase in levels of TNF, transforming growth factor (TGF), activation of liver sinusoidal endothelial cells, and hepatocyte chromosomal aberrations [172]. Overproduction of ROS is triggered by increased fatty acid oxidation and hepatocyte metabolism [173]. Excess in triglycerides and free fatty acids (FFAs) prevents autophagy by activating the mammalian target of rapamycin (mTOR). DNA damage and oxidation occur when the antioxidant limit of the hepatocytes is crossed, ultimately leading to cell death [181, 182].

### Lifestyle Risk Factors

Alcoholic liver disease (ALD) is a severe disease that affects hepatic metabolism and leads to steatosis, fibrosis, cirrhosis, and liver cancer [183, 184]. Alcohol intake as low as 10 g a day raises the risk of HCC, which accounts for 30% of all liver cancer deaths worldwide [185]. Fatty acid oxidation and lipogenesis are two metabolic pathways that are affected by alcohol consumption [186]. Alcoholic steatohepatitis enhances the production of HCC by up-regulating inflammation, cell proliferation, and mir-122 loss. Genetic variations on alcohol metabolizing enzymes resulting in the accumulation of acetaldehyde (carcinogenic) and thus serves as potential inheritable HCC markers. Alcohol-induced oxidative stress leads to the formation of ROS.its buildup has structural and functional effects on DNA, resulting in cell cycle arrest and death. Alcohol disrupts the synthesis of S-adenosyl-L-methionine (SAMe) and methylation status, all of which are related to the growth of HCC. It is worth noting that the magnitude of ALD is related to genetic susceptibility. PNPLA3 and MBOAT7/TMC4 have also been linked to higher chances of cirrhosis in alcoholics [187].

### Environmental Carcinogens

HCC carcinogenesis is linked to a variety of environmental chemicals. Aflatoxin is the most well-known among these. Other factors are exposure to vinyl chloride, arsenic, polycyclic hydrocarbons, and radioactive compounds [188]. Aflatoxins are mycotoxins formed by the fungi *Aspergillus flavus* and *Aspergillus parasiticus* and are commonly found in infected grain products like corn, peanuts, and legumes [189, 190]. Aflatoxin B1 causes carcinogenesis in animals and humans by forming DNA adducts with hepatic DNA [191]. In areas with high aflatoxin exposure, as much as a 70-fold increase in chances of HCC has been recorded [192].

## Conclusion

Liver cancers, particularly HCC, strongly affect human health worldwide. Unfortunately, despite the advancements in cancer diagnostics and therapeutics, we have not reached the potential to overcome such disease conditions without side effects effectively. Thus, advanced alternative therapies are needed that can overcome such shortcomings. The *C. longa* and current treatments can be used because they have high antioxidant and anti-inflammatory properties that can help limit human HepG2 cells development. Curcumin has been successfully used with other treatment options like leflunomide, perindopril, and similar antiangiogenic agents to treat HCC [192]. In addition, curcumin has shown the power to affect several signaling pathways, suggesting its antitumor potential. Thus, curcumin might be a good option for preventing oxidative stress-related liver disease by lowering ALT, AST, and ALP levels.

## Future Prospective

Furthermore, curcumin, a herb, shows insignificant side effects and is safer and inexpensive than other medicines and therapies. Thus, apart from showing synergistic anticancer effects, it also protects from the potential side effects of chemotherapies. However, there are a few shortcomings to this therapy that still needs to be addressed. For starters, curcumin has low bioavailability, instability, hydrophobicities, and the ability to rapidly clear from the body, hindering its practical usage in clinical applications and as anticancer therapy. This disadvantage can be addressed by functionalizing curcumin with nanomaterials, microcells, liposomes, phospholipid complex before being used as therapeutics. Therefore, further study must be done to understand curcumin’s chemical kinetics and dynamic action and analogs, better analyze curcumin’s therapeutic strategy, and determine the appropriate curcumin dose required for effective HCC treatment.

Thus, the recent findings imply that *C. longa* is a viable natural product source and promising candidate to be used as an adjuvant in treating HCC. This molecule can be developed into a novel anticancer medicine once its complete apoptotic action has been studied clinically.

## Data Availability

All data are given in the manuscript.

## Abbreviations

HCC: Hepatocellular carcinoma
HCV: Hepatitis C virus
NASH: Nonalcoholic steatohepatitis
HBV: Hepatitis B virus
NAFLD: Nonalcoholic fatty liver disease
ALD: Alcoholic-related liver disease
CHB: Chronic hepatitis B
CDC: Centers for Disease Control and Prevention
GCC: Gulf Cooperation Council
DMBA: Dimethylbenz(a) anthracene
AESN: Aqueous extracts of *Solanum nigrum*
ACE2: Angiotensin-converting enzyme 2
CCL4: Carbon tetrachloride
STAT3: Signal transducer and activator of transcription 3
CDK: Cyclin-dependent kinase
HepG2: Hepatoma G 2
ROS: Reactive oxygen species
BCL2: B-cell lymphoma 2
VEGF: Vascular endothelial growth factor
NF-KB: Nuclear factor Kappa light chain enhancer of activated B cells
AP-1: Activator protein 1
PI3K: Phosphoinositide 3-kinase
ALT: Alanine aminotransferase
AST: Aspartate aminotransferase
ALP: Alkaline phosphatase
SOD: Superoxide dismutase
GSH: Glutathione
GR: Glutathione reductase
MDA: Molanodialdehyde
CAT: Catalase
iNOS: Inducible nitric oxide
HDL: High-density lipo-protein
LDL: Low density lipo-protein
TG: Triglyceride
ERK: Extracellular signal-regulated kinases
MAPK: Mitogen-activated protein kinase
PARP: Polyadenosine diphosphate ribose polymerase
TNFα: Tumor necrosis factor-alpha
MCP-1: Monocyte chemoattractant protein-1
CRP: C-reactive protein
PGE2: Prostaglandin E2
COX: Cyclo-oxygenase
NOS: Nitric-oxide synthase
PERK: Protein Kinase-like endoplasmic reticulum kinase
p-PERK: Phosphor-thr982
IRE1 α: Inositol-requiring enzyme 1 alpha
PGCs: Primordial germ cells
CTCs: Circulating tumor cells
*NADH*: Nicotinamide adenine dinucleotide
NO: Nitric oxide
TACE: Trans-arterial chemoembolization
HbsAg: Hepatitis B virus surface antigen
HBX: Hepatitis B virus X-protein
NS3: Nonstructural protein 3
NS5A: Nonstructural protein 5A
RBI: Retinoblastoma protein
TGF: Transforming growth factor
mTOR: Mammalian target of rapamycin
mir-122: MicroRNA-122
PNPLA3: Patatin-like phospholipase domain-containing protein-3
TMC4: Transmembrane channel-like protein 4
AfB1: Aflatoxin B1
MBOAT7: Membrane-bound o-acyltransferase domain-containing protein7
